# Advances in Optical Tissue Clearing for Three‐Dimensional Ocular Imaging and Analysis

**DOI:** 10.1155/joph/2810267

**Published:** 2026-04-01

**Authors:** Shuhuai Meng, Haiyang Sun, Zhengyi Xu, Yixin Shi, Mingyang Chen, He Cai, Junyu Chen

**Affiliations:** ^1^ West China Hospital, Sichuan University, Chengdu, 610041, China, scu.edu.cn; ^2^ State Key Laboratory of Oral Diseases, National Clinical Research Center for Oral Diseases, West China Hospital of Stomatology, Sichuan University, Chengdu, 610041, China, scu.edu.cn; ^3^ Department of Stomatology, West China Tianfu Hospital, Sichuan University, Chengdu, 610200, China, scu.edu.cn

**Keywords:** eye tissues, microscopy, ophthalmology, tissue clearing methods, vascular and neural architecture

## Abstract

The visualization of tissue architecture has long been constrained by optical absorption and scattering phenomena. Recent advances in optical tissue clearing techniques have revolutionized deep tissue imaging by overcoming these fundamental limitations, enabling high‐resolution microscopic examination of intact organ systems. In ophthalmic research, these methodologies provide transformative capabilities for macroscopic three‐dimensional visualization, effectively addressing the spatial constraints inherent to conventional two‐dimensional histological sections. This paradigm shift has opened new avenues for investigating complex spatial relationships in ocular biology, including vascular network organization, neural connectivity patterns, and dynamic cellular processes. This comprehensive review synthesizes the current literature on tissue clearing methodologies with specific emphasis on ocular applications, systematically examining (1) the unique structural considerations for eye tissue clearing, (2) the established protocols for ocular tissue clearing, (3) the new discoveries in ocular structures through tissue clearing, (4) the imaging acquisition and analysis for cleared eye tissue, and (5) the emerging directions for future technological development. By integrating diverse insights, this review establishes a foundation for the continued refinement of tissue clearing approaches in ocular research, promoting a deeper understanding of eye structure and pathology.

## 1. Introduction

The human eye, although only a small portion of the body, serves as an indispensable window into systemic health and neurological integrity. Emerging evidence has established many correlations between ocular structural alterations and neurodegenerative pathologies including Alzheimer’s disease [1–3], Huntington’s disease [4, 5], Parkinson’s disease [6, 7], and related disorders. Recent discoveries reveal that detailed features of ocular vascular structure, neural networks, and protein deposition patterns may provide key insights for the diagnosis and treatment of cerebrovascular and cardiovascular diseases.

To explore the eye’s intricate microstructures, scientists have sought advanced imaging approaches. Techniques such as positron emission tomography [8] and magnetic resonance imaging (MRI) are commonly used to assess tissue pathology, but their spatial resolution is insufficient to detect subtle submillimeter changes. Although traditional tissue sectioning methods can observe at micrometer thickness (5–10 μm), the imaging depth is limited and inherently destroys the three‐dimensional integrity of the tissue. In clinical ophthalmology, imaging modalities such as fundus photography, fluorescein angiography, and optical coherence tomography angiography (OCTA) are routinely employed to assess microvascular damage. However, these technologies are limited by hardware limitations and cannot provide accurate reconstruction of eye tissue volume [9–11]. In addition, the spherical geometry of the eye can cause imaging artifacts due to continuous slicing or tiling processing.

Tissue clearing has emerged as a powerful alternative for histological analysis, offering a non‐destructive approach to deep tissue imaging. By rendering optically transparent and reducing refractive heterogeneity, tissue clearing methods allow light to penetrate deeply without the need for slicing. This preserves native tissue architecture and enables comprehensive 3D visualization of complex biological structures, greatly enhancing the precision of spatial and molecular analyses.

Despite these advantages, the eye presents unique challenges for tissue clearing due to its multilayered composition and significant refractive index variations in different eye compartments. As a result, standard clearing protocols often require some modification for ocular applications. The latest progress in clearing methods now enables high‐resolution and accurate 3D visualization of tissue structures, opening up new avenues for ophthalmic research and disease modeling [12–14].

This review summarizes the latest developments in tissue clearing techniques as applied to the eye. We outline the principles and protocols involved in achieving optical transparency of ocular tissues and highlight their applications in basic research. Through more precise qualitative analysis of proteins, subcellular components, and microscopic anatomical structures, tissue clearing is reshaping the landscape of vision science and neuropathological research.

## 2. The Unique Challenges of Ocular Tissue Structure in Tissue Clearing

The eye is composed of two primary anatomical components: the eyeball wall and intraocular contents. Although the eyeball wall is thin, it possesses a remarkably complex organization, including three distinct layers: (1) the innermost retina, (2) the middle choroid, comprising the iris, ciliary body, and choroid proper, and (3) the outer fibrous layer, composed of the cornea and sclera. The inner contents of the eyeball include the aqueous humor, vitreous humor, and lens. While a variety of tissue clearing techniques have been successfully applied to soft and hard tissues, the unique structural and optical properties of the eye pose significant challenges for achieving true transparency (Table 1). Specialized protocols are required for different components of the eye due to their diverse biochemical compositions and refractive heterogeneities.

**TABLE 1:** Key processing steps for transparency in the eyeball.

Eye tissue component	Light‐blocking components	Special processing steps	Notes
Fibrous layer	Collagen fibers	Low‐temperature dehydration, chemical crosslinking, use of high‐permeability solutions	Reduce collagen porosity, maintain structural stability
Choroid and retina	Pigment cells (melanin)	H_2_O_2_ bleaching (3%–15%, 4°C–65°C)	Optimize concentration/time to avoid protein damage and autofluorescence
Contents	—	—	Refractive index matching

### 2.1. The Fibrous Layer: Collagen‐Dense Tissue That Resists Transparency

**FIGURE 1:**
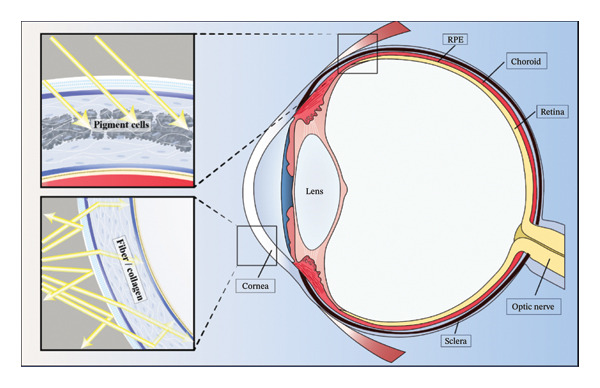
Structural features contributing to ocular tissue opacity. Key determinants of optical transparency in ocular tissues: (upper left) pigment cells absorb incident light; (bottom left) fiber or collagen networks induce light scattering.

The fibrous outer layer, consisting of the cornea and sclera, is primarily composed of dense, organized collagen fibers. Under physiological conditions, the cornea is naturally transparent due to the highly ordered arrangement and low hydration of its collagen matrix. However, pathological changes such as corneal edema, inflammation, or ulceration can disrupt this order and lead to opacity (Figure 1). Achieving optical clarity in this layer is challenging due to its dense fibrous composition. Studies have shown that low‐temperature dehydration combined with wet chemical crosslinking can induce transparency in collagenous tissues by reducing light scattering [15]. Additionally, the application of highly permeable dehydrating agents has proven effective in extracting water while preserving collagen structure [16]. The key to clearing the fibrous layer lies in minimizing collagen porosity and maintaining fibrillar integrity, which helps reduce refractive index mismatches and scattering.

### 2.2. The Choroid and Retina: Pigment‐Rich Layers That Inhibit Light Transmission

The choroid and retinal pigment epithelium (RPE) are densely populated with pigment cells, particularly melanocytes, which strongly absorb and scatter light. These pigmented layers severely hinder light transmission and are a primary cause of tissue opacity in the eye (Figure 1). Unlike other organs, where standard clearing protocols can render tissue transparent, ocular pigment presents a significant barrier to achieving clarity [17, 18]. Therefore, effective pigment removal is a critical step in eye tissue clearing. Hydrogen peroxide (H_2_O_2_) is commonly used as a bleaching agent; however, excessive concentrations or prolonged exposure can damage proteins and disrupt tissue architecture [19]. To mitigate these effects, protocols have been optimized for H_2_O_2_ concentration (typically 3%–15%), bleaching temperature (4°C–65°C), and incubation time [19, 20]. Despite these improvements, Nazari et al. cautioned that oxidative bleaching may introduce autofluorescence and quench endogenous fluorophores, compromising downstream imaging [21]. As a result, mechanical removal of pigmented tissue is sometimes preferred, especially for studies requiring high‐fidelity fluorescence imaging [21].

### 2.3. Intraocular Contents: Transparent Structures That Still Require Structural Context

The contents of the eyeball—aqueous humor, vitreous humor, and lens—are generally transparent under normal physiological conditions and play a direct role in visual function. However, they do not exist in isolation. The vitreous body, in particular, is surrounded by vascular and neural networks that are crucial in diseases such as retinopathy of prematurity, Norrie disease [22], and Stickler syndrome [23]. Therefore, even though the intraocular media themselves do not require clearing, it is essential to preserve and visualize the surrounding structures to reconstruct accurate three‐dimensional anatomical and pathological contexts.

Understanding the specific characteristics of each ocular component is fundamental to developing and refining tissue clearing workflows for the eye. Tailored protocols that consider the biochemical and optical properties of each layer ensure successful clearing outcomes and enable high‐resolution imaging across the entire globe.

## 3. Methods for Clearing Eyeball Tissue

Recent advances in imaging technologies have significantly enhanced our ability to visualize and analyze the three‐dimensional architecture of ocular vascular systems. To obtain comprehensive volumetric information from eye tissues, numerous tissue clearing protocols have been developed, predominantly through modifications of the existing whole‐organ clearing methodologies. Each ocular tissue clearing approach features distinct processing workflows and utilizes specialized chemical reagents (Table 2). Based on the delipidation properties of clearing agents, tissue clearing techniques are categorized into three types: tissue transformation‐based methods, high‐refractive index aqueous matching methods, and organic solvent–based clearing methods.

**TABLE 2:** Optical tissue clearing techniques for eyeball clearing.

Clearing strategy class	Method	Clearing principle	Fluorescence preservation	Tissue distortion	Vitreous preservation	Typical processing time	Representative ophthalmic applications
Tissue transformation based	CLARITY	Hydrogel embedding, electrophoretic lipid removal	Excellent	Minimal	Moderate	10–14 days	Retinal neurons, glial networks, optic nerve architecture
	PACT	Hydrogel embedding, passive lipid extraction	Excellent	Minimal	Moderate	5–7 days	Retinal vascular network, whole eyeball imaging

High‐refractive index aqueous matching based	SeeDB	Fructose‐based RI matching	Excellent	Negligible	Poor	2–4 days	Sclera, cornea morphology preservation
	CUBIC	Urea/Quadrol‐based delipidation, bleaching, and RI matching	Good	Swelling	Moderate	1–2 weeks	Whole eyeball imaging, retinal vasculature

Organic solvent–based	BABB	Ethanol dehydration, BA/BB RI matching	Poor	Significant shrinkage	Poor	1–2 days	Sclera, retinal vasculature
	DISCO series (3DISCO, iDISCO, iDISCO+)	THF‐based dehydration, delipidation, DBE RI matching	Moderate–good	Pronounced shrinkage	Poor	3–7 days	Whole eyeball imaging, optic nerve
	EyeDISCO/EyeCi	H_2_O_2_ bleaching, dehydration, delipidation, RI matching	Good	Moderate shrinkage	Moderate	5–7 days	Retina‐vitreous‐uvea vascular networks
	SHANEL	CHAPS/NMDEA micelle‐mediated permeabilization, dehydration and delipidation, RI matching	Good (endogenous fluorescent protein signals)	Moderate shrinkage	Poor	Several weeks	Whole human eyeball imaging
			Poor (immunofluorescence)				
	PEGASOS/TESOS	tB/PEG‐based dehydration and delipidation, BB‐PEG RI matching	Excellent	Severe shrinkage	Poor	5–7 days	Whole eyeball imaging

### 3.1. Organic Solvent–Based Clearing Methods

Organic solvent–based clearing methods utilize water‐immiscible organic solvents to render tissues transparent through dehydration, delipidation, and refractive index matching.

#### 3.1.1. BABB Series Techniques

The benzyl alcohol/benzyl benzoate (BABB) method remains a widely used clearing approach due to its simplicity, rapid processing, and cost‐effectiveness. This protocol involves graded ethanol dehydration followed by immersion in a 1 : 2 BA : BB solution [24]. Waxman et al. achieved tissue transparency in pig eye tissue using the BABB method. Their research revealed that the tissue clearing approach with BABB yielded superior transparency results in scleral tissue compared to the clear, unobstructed brain imaging cocktails and computational analysis (CUBIC) series [25]. BABB has also proven valuable for corneal biomechanics research, enabling qualitative assessment of stiffness alterations in porcine corneas [26]. A major limitation of the BABB technique is that its organic solvents rapidly quench fluorescence and fail to effectively clear highly myelinated tissues such as the optic nerve. However, BABB has proven to be effective in preserving the signal of lactin conjugates. Lectin‐FITC has been proven to be highly effective as a fluorescent marker for retinal vascular endothelium [27–29]. Therefore, the BABB technique offers unique advantages in investigating changes in retinal vasculature.

#### 3.1.2. SHANEL Series Techniques

SHANEL [30], as a classic tissue clearing technique, primarily utilizes CHAPS (3‐[(3‐cholamidopropyl) dimethylammonio]‐1‐propanesulfonate) and NMDEA (N‐methyldiethanolamine) to process tissue samples. This method forms small micelles that can completely diffuse through intact large mammalian organs, improving tissue reticulation while leaving behind fully permeable biological specimens. The technique has successfully achieved transparency in human ocular tissues, enabling the acquisition of complete eyeball TO‐PRO‐3 and endogenous fluorescent protein signals.

#### 3.1.3. Dimensional Imaging of Solvent‐Cleared Organs (DISCO) Series Techniques

The DISCO series has emerged as a cornerstone technique for eyeball tissue clearing, with its foundation rooted in the BABB methodology. The original 3DISCO protocol introduced key innovations by substituting traditional alcohol dehydration with tetrahydrofuran (THF), which better preserves fluorescence signal intensity, while employing dibenzyl ether (DBE) for efficient lipid removal [31]. Subsequent refinements have yielded several specialized variants, with iDISCO and iDISCO+ proving particularly valuable for ocular applications. The iDISCO technique incorporates H_2_O_2_‐mediated bleaching to eliminate endogenous pigments, achieving exceptional transparency in optic nerve tissues [32, 33]. Building upon this, iDISCO+ addresses THF‐related limitations (including peroxide formation and YFP signal loss) through the addition of dichloromethane and methanol, which reduce immunostaining background and minimize tissue dehydration [34]. Collectively, this approach provided high‐resolution imaging of the intricate spatial architecture of capillary networks within the intact eyeball, exemplified by Darche et al.’s successful whole‐eye clearing and high‐resolution imaging of capillary networks [14]. To ensure robust fluorescence protein signals and achieve high‐quality transparency in eye tissue, a method called “EyeCi” combines the techniques of “Ethanol‐Eci” and iDISCO+. This approach effectively achieves tissue transparency in the eye. Subsequently, target proteins are labeled by injecting antibodies into the eye, leading to a well‐preserved structure of the mouse retinal vascular network [35]. Vigouroux et al. [36] developed an enhanced method called EyeDISCO, building upon the foundation of iDISCO+. This method primarily involves modifications to the endogenous pigment bleaching technique targeting the RPE. Due to the issue of spontaneous fluorescence generated by organic solvent‐based clearing, choosing photobleaching can reduce spontaneous fluorescence in thick tissue samples. EyeDISCO employs an 11% H_2_O_2_ solution for bleaching and utilizes LED lights with a color temperature of 3000 K and an 11 W illumination. Following the bleaching step, the cornea is incised to facilitate subsequent immunofluorescence staining. The EyeDISCO protocol enables high‐resolution volumetric imaging of the complete vascular networks in the retina, vitreous, and uvea. This approach captures the dynamic remodeling of the iris vasculature, including sprouting and pruning events, following its developmental disconnection from the embryonic hyaloid circulation system [13]. The newest wildDISCO variant further enhances antibody penetration through cyclodextrin‐mediated cholesterol extraction, proving valuable despite the eye’s relatively low cholesterol content [37]. Furthermore, several modified DISCO‐based whole‐body clearing methods have demonstrated the ability to achieve transparency in ocular tissues [38, 39]. While DISCO methods excel at revealing optic nerves, retinal ganglion cells (RGCs), and ocular tissue architecture, users must consider their limitations: potential tissue shrinkage affecting morphometric analyses, the high toxicity of THF/DBE reagents, and possible compromise of endogenous fluorescent protein signals [40]. These factors necessitate careful protocol selection based on specific experimental requirements.

#### 3.1.4. PEGASOS Series Techniques

PEGASOS [18] and its derivative method TESOS [41] employ tB (tert‐Butanol)/PEG (polyethylene glycol) derivatives and dental composite resin monomers (bisphenol A ethoxylate diacrylate, BED) combined with high‐refractive‐index benzyl benzoate [42] as refractive index matching solutions, respectively. PEGASOS technology can effectively and transparently remove almost all tissues except for the RPE, with a 6‐day preservation rate of endogenous fluorescence of approximately 70%. Its disadvantage lies in tissue shrinkage, with a shrinkage rate of 30%–40% [18]. TESOS technology makes eye tissue samples highly transparent. It enables nearly damage‐free 3D imaging of biological samples. This method can image large volumes with high precision. It achieves micron to submicron resolution in all directions. After solidification, samples become very hard. Cutting along the *Z* axis hardly affects the continuity of the final 3D image depth [41].

Although organic solvent‐based clearing methods exhibit excellent transparency and enable subcellular‐resolution imaging of ocular tissues, they also have several limitations. For example, the dehydration steps involved in the clearing process can lead to substantial sample shrinkage. In addition, most organic solvents are toxic, and these reagents can cause quenching of fluorescent proteins.

### 3.2. High‐Refractive Index Aqueous Matching Methods

High‐refractive index aqueous matching methods, based on water‐soluble reagents, exhibit high biocompatibility and safety, preserving protein function. They achieve transparency through processes such as defatting, decoloring, refractive index matching, and (optional) swelling. Some methods allow for tissue swelling to enhance resolution, but may require longer incubation times.

#### 3.2.1. See Deep Brain (SeeDB) Series Techniques

The SeeDB technique uses fructose‐based refractive index matching for tissue clearing [43]. While effective for scleral transparency in human and avian eyes, SeeDB fails to clear pigmented retinal epithelium [44]. Its key advantage is minimal tissue volume alteration, crucial for preserving delicate ocular structures.

#### 3.2.2. CUBIC Series Techniques

The CUBIC primarily utilizes two mixed solutions for tissue clearing. The mixed solution CUBIC1, consisting of urea, Quadrol, and Triton X‐100, is used for bleaching and delipidation of tissue samples. The mixed solution CUBIC2, composed of urea, sucrose, and triethanolamine, is employed for tissue transparency matching the refractive index [45, 46]. While Quadrol serves as the primary bleaching agent, its efficacy is limited for pigmented ocular structures such as sclera and RPE [46]. Ye et al. addressed this limitation by implementing a 55°C 10% H_2_O_2_ pretreatment, achieving improved tissue transparency through optimized CUBIC 1/CUBIC 2 incubation times [47]. However, complete retinal vascular network visualization remains challenging, potentially due to freeze–thaw‐induced vascular damage. Other limitations include prolonged processing times, poor antibody penetration through scleral barriers, and clearing solution evaporation affecting imaging quality [48].

### 3.3. Tissue Transformation‐Based Methods

This process is a tissue clearing method based on hydrogel, which embeds and fixes biological molecules to maximize the preservation of tissue structure and component integrity.

The CLARITY (Clear Lipid‐exchanged Acrylamide‐hybridized Rigid Imaging compatible Tissue hYdrogel) method represents a hydrogel‐based tissue clearing approach. This technique involves the infusion of hydrogel monomers into tissues under a nitrogen atmosphere, creating covalent bonds between biomolecules (proteins, nucleic acids, and small molecules) and the hydrogel matrix through formaldehyde‐mediated crosslinking. Subsequent electrophoretic lipid removal renders tissues optically transparent [49]. Application of CLARITY to murine retinal tissues has enabled three‐dimensional visualization of neuronal networks, glial cells, and synaptic proteins [50]. Protocol modifications, including adjustments to acrylamide concentration and solution pH, have further improved retinal vascular imaging [51, 52]. Yang et al. enhanced pigment removal by incorporating a 55°C 15% H_2_O_2_ bleaching step prior to CLARITY processing, with the X‐CLARITY automated system significantly improving both processing efficiency and tissue preservation [53].

**FIGURE 2:**
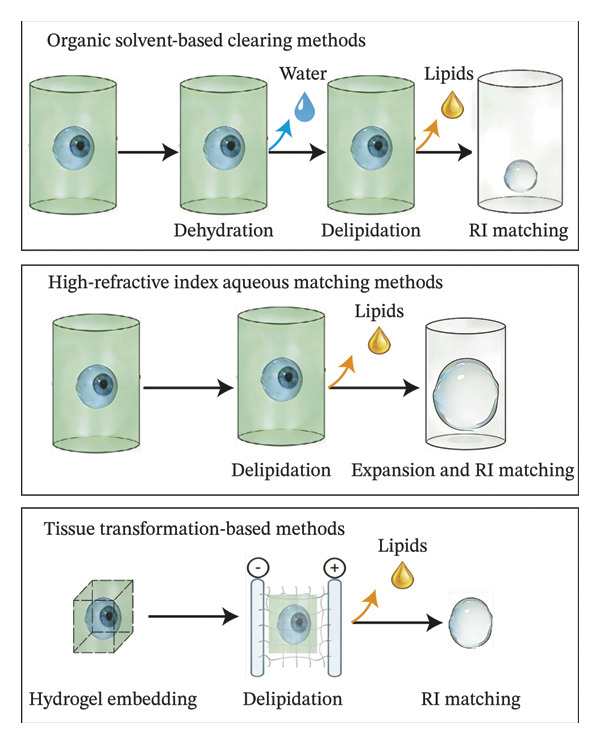
Comparative workflow of the three major clearing methodologies: organic solvent‐based clearing methods, high‐refractive index aqueous matching methods, and tissue transformation‐based methods.

PACT (Passive Clearing Technique) addresses several CLARITY limitations through optimized hydrogel composition and clearing reagents. By reducing hydrogel density and crosslinking, PACT enhances tissue permeability, enabling effective passive lipid extraction from 1‐ to 3‐mm‐thick specimens [54]. This modification proves particularly suitable for small organs such as mouse eyes, where hydrogel infiltration protects delicate retinal structures while maintaining vascular network integrity [55, 56]. Moreover, PACT enabled prolonged preservation of fluorescence signals [55]. Various clearing methods exist with distinct protocols but shared principles. No universal method suits all applications. Optimal results require method selection based on (1) research objectives, (2) ocular tissue properties, and (3) fluorescent antibody compatibility. Suboptimal choices may compromise outcomes. Figure 2 illustrates the key steps and time requirements of these four primary eyeball tissue clearing methods.

## 4. New Insights Into Ocular Structures Through Tissue Clearing

### 4.1. Breakthroughs in Eyeball’s 3D Vascular Network

The intricate ocular vascular system serves not only as a critical component in numerous blinding diseases but also represents an exemplary model for studying angiogenesis. Recent advances in tissue clearing techniques have enabled unprecedented three‐dimensional visualization and quantitative analysis of this complex vascular network, which involves the coordinated development of multiple interconnected systems. By combining iDISCO+ with light‐sheet fluorescence microscopy (LSFM), Darche and colleagues achieved the comprehensive 3D reconstruction of murine ocular vasculature [57]. Their work provided novel insights into the spatiotemporal relationship between hyaloid vessel regression and retinal vascular development, while also precisely mapping collateral vein formation in branch retinal vein occlusion. Yin et al. discovered a unique lymphatic drainage system in the posterior eye (Figure 3(a)), which is crucial for eye–brain immunity and offers new treatment strategies for related diseases [58].

FIGURE 3Novel discoveries into ocular structures through tissue clearing. (a) Visualization of the lymphatic drainage system in the posterior eye using tissue clearing. Representative images (left panel) of optic nerve and chiasma, lymphatics stained with LYVE1 and VEGFR3 (white arrows). Representative images (right panel) of optic nerve sheaths stained for LYVE1, CD31, PROX1, and VEGFR3. Scale bar: Left panel, 3000 μm; right panel, 500 μm. Reproduced with permission from Yin et al., Nature, 628 (8006): p. 204–‐211 [58]. Copyright 2024 by Springer Nature. (b) EyeDISCO tissue clearing showing the critical role of deleted in colorectal carcinoma (DCC) in ocular development. (A) Workflow diagram of the EyeDISCO. (B, B′) Representative images of a whole mouse eye prior to and after tissue clearing. (C, D) Fundoscopy reveals severe dysplasia in Dkk3: cre; Dccfl/fl eyes (yellow arrows) versus controls (Dccfl/fl). Eye axes: D (dorsal), V (ventral), N (nasal), T (temporal). (E, F) Cleared whole‐mount eyes (TO‐PRO‐3 nuclear stain) showing dysplasia (yellow arrows). (G, H) 3D reconstruction (Imaris) of dysplastic regions (magenta) in Dkk3: cre; Dccfl/fl eyes. (I–N) Rosette structures in the bilateral eyes of three mutant mice. (O–P) Whole‐mount IHC for short‐wavelength opsin (Opn1sw) in control and mutant eyes. (Q) Merged 3D masks of rosette areas (magenta) and retinal circumference. (R) Quantification showing no significant difference in rosette coverage between Dcc cKO and controls across ages. Scale bars: 300 μm (E–H, I–N), 500 μm (O–P), 150 μm (O–P, high magnification), 400 μm (Q). Reproduced with permission from Vigouroux et al., Elife, 9 [36] copyright 2020 by eLife.

**Figure:**
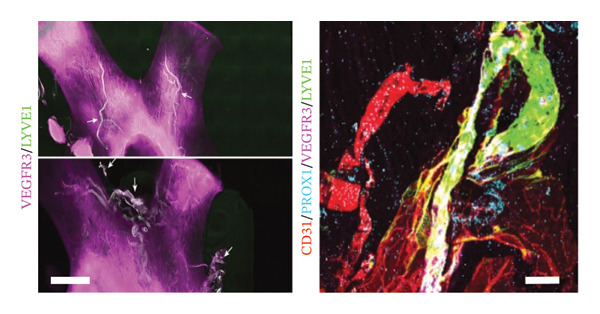
(a)

**Figure:**
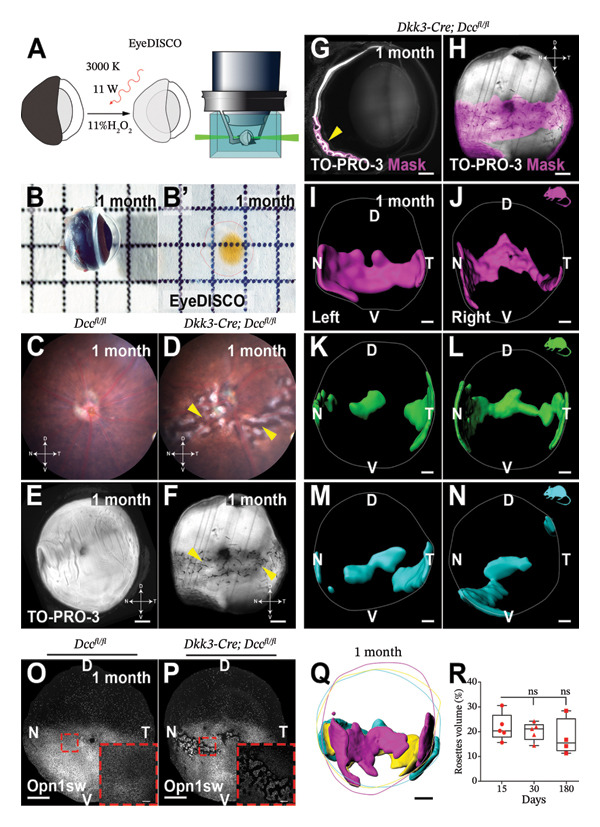
(b)

### 4.2. Novel Discoveries in Ocular Structural Disorders

The integration of tissue clearing techniques with LSFM has revolutionized three‐dimensional reconstruction of ocular tissues, opening new avenues for investigating disease pathogenesis and developing therapeutic interventions. Prahst et al. utilized the PACT method to characterize endothelial cell dynamics during ocular angiogenesis, revealing abnormal cellular migration patterns in oxygen‐induced retinopathy (OIR) models. Their work not only identified unique structural features of pathological vascular tufts in OIR, but also enabled simultaneous visualization of retinal neurons and vascular networks [55]. In another significant contribution, Vigouroux et al. applied EyeDISCO to demonstrate the critical role of deleted in colorectal carcinoma (DCC) in ocular development and maintenance (Figure 3(b)). Their findings showed that DCC deficiency disrupts RGC axonal guidance, leading to optic nerve hypoplasia, while also being essential for the survival of both RGCs and photoreceptors [36]. Further advancing the field, Lu et al. optimized the iDISCO protocol to achieve comprehensive 3D mapping of RGC central projections. They found that acute ocular hypertension (AOH) damages specific RGC populations, particularly parvocellular‐projecting cells, offering new insights into selective neurodegeneration and guiding future therapies [12]. Similarly, using the iDISCO+ protocol, Cooper and colleagues found that early‐stage glaucomatous optic neuropathy manifests as impaired axonal transport in RGCs and altered astrocyte function. In a mouse model, they observed that RGC projections in the accessory optic tract were the first to lose transport function, and unilateral glaucoma could trigger widespread astrocyte reactivity in both cerebral hemispheres [59].

Tissue clearing technology is revolutionizing ophthalmic medicine by transforming traditional two‐dimensional diagnostics into comprehensive three‐dimensional assessments. This technological advancement enables a systemic‐level understanding of ocular diseases, creating unprecedented opportunities for early detection and intervention in previously untreatable blinding conditions.

## 5. Organ‐Level Imaging Acquisition and Analysis for Cleared Eye Tissue

Cleared eyes are typically immersed in clearing solutions for preservation and imaging. After undergoing tissue clearing, eye tissue not only allows for easy acquisition of horizontal axis data but, more importantly, enables the acquisition of longitudinal data. To obtain high‐resolution three‐dimensional imaging information, it is crucial to select imaging devices with a sufficient working distance based on the tissue thickness. This allows for the study and analysis of retinal vascular network changes with temporal and spatial heterogeneity that cannot be explored in 2D imaging alone [60]. Furthermore, it enables research and analysis of lymphatic vessels, nerve plexus, and drainage pathways within the eyeball under pathological conditions.

### 5.1. LSFM

LSFM, also known as light‐sheet microscopy, is currently the most commonly used imaging device for tissue clearing [61–63]. It possesses optical sectioning capability by creating a thin sheet of light emitted from the side of the sample, generating a plane of light within the tissue without exciting fluorescent molecules outside of that plane. This feature enables LSFM to have extremely low phototoxicity and photobleaching [64]. Due to the fact that LSFM scans the sample using a light plane rather than point scanning, the image acquisition speed of LSFM is exceptionally fast. In recent years, LSFM has widely been used for imaging observations in eye tissue after tissue clearing. It only takes approximately 1 min to obtain a complete visualization of the retinal vascular structure [51, 52, 55]. LSFM enables the comprehensive capture of the complete three‐dimensional interconnected network of blood vessels, lymphatic vessels, and other structures within the eye. It also allows for three‐dimensional imaging of different types of cells and subcellular structures within the eye (Figure 4). In the retina, vascular development is regulated by a series of signaling pathways that are coordinated by environmental cues and cellular metabolic activities [60, 65]. The LSFM imaging system provides a reliable platform for studying pathological damage, regeneration, and protein–molecular interactions within the retinal vascular structure.

FIGURE 4LSFM imaging of cleared ocular vasculature. (a) Imaging of the retinal vasculature. (A, B) Representative images of retinal vasculature showing regions of interest. (C) 2D section (red dashed lines in (B)) highlighting vertical sprouts connecting primary and secondary vascular plexuses. (D) Higher magnification insets (blue box in B) show retinal vasculature. (E, F) Higher magnification insets (orange box in B) showing 3D (E) and 2D (F) views of peripheral retina. (G, H) Capillary network analysis: maximum intensity projection (G) and depth color‐coding (H, 0–240 μm) of the region in (F). Scale bars: 500 μm (A–C), 100 μm (D), 300 μm (E, F), and 50 μm (G, H). Reproduced with permission from Chang et al., Theranostics, 11 (16): p. 1162–‐1175 [51]. Copyright 2021 by Ivyspring International Publisher. (b) Superimposed hyaloid and retinal vasculatures of eyes. Retina vasculatures in cyan; hyaloid arteries in magenta. Scale bars: 500 μm. Reproduced with permission from Darche et al., Commun Biol, 5 (1): p. 1135 [57]. Copyright 2022 by Springer Nature.

**Figure:**
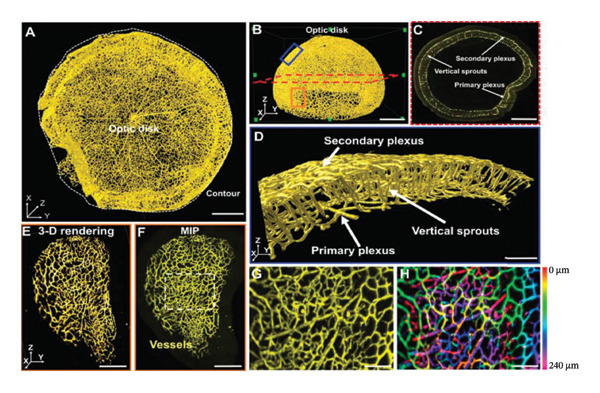
(a)

**Figure:**
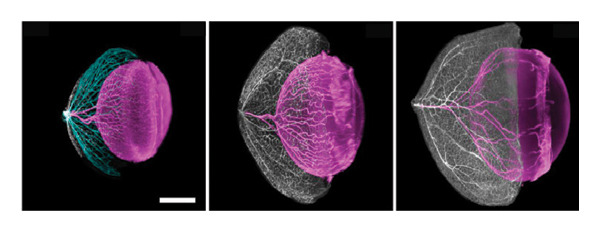
(b)

### 5.2. Confocal Laser Scanning Microscope (CLSM)

CLSM is a powerful optical imaging device widely used for visualizing cells and subcellular structures within biological tissues at a high resolution [66, 67]. One of the primary advantages of CLSM is its capability to acquire high‐quality images with high spatial resolution in the micrometer range and high contrast. CLSM scans tissue samples using single or multiple focal points, resulting in a slower imaging speed compared to LSFM. Based on our experience, imaging the entire rat retina using a 10× objective lens with CLSM typically takes around 2 h. The imaging depth of a CLSM system can reach up to 1000 μm, enabling easy observation of structures such as the sclera, retina, and optic nerve [25, 36].

### 5.3. Two‐Photon Microscope (TPM)

TPM is an imaging system that combines laser scanning confocal microscopy with two‐photon excitation technology [42]. When using TPM to observe samples, fluorescent molecules located outside the focal plane are not excited, leading to reduced phototoxicity and photobleaching. This makes it well‐suited for imaging with minimal sample damage. Moreover, TPM offers superior tissue penetration depth compared to confocal microscopy. In a study conducted by Lee et al. on corneal neovascularization [68], it was observed that the irregular and heterogeneous structure of neovascularized cornea causes optical aberrations and scattering, posing challenges for identifying corneal cells undergoing neovascularization using confocal microscopy. However, TPM, employing longer excitation wavelengths and collecting signals from scattered emitted light within the sample, exhibits lower sensitivity to light scattering. This enables TPM to capture a greater amount of signals from deeper tissue regions.

Tissue clearing and imaging are interdependent processes that work together to provide three‐dimensional visualization of deep structures within the eye. The progress in optical imaging systems has provided significant improvements in terms of high axial resolution, wide field of view, and rapid imaging speed. When coupled with tissue clearing techniques, these advancements empower us to achieve a comprehensive understanding of eye tissue, offering an ideal platform for investigating the developmental processes and pathological changes that take place within the eye.

### 5.4. Three‐Dimensional Image Data Analysis for Cleared Eye Tissue

With the advent of tissue clearing techniques and powerful imaging microscopy devices, researchers can now access a wealth of information from within tissues. To effectively store, process, and extract biological significance from the acquired image data, as well as to tackle the complexity of the retinal vascular network and cellular protein composition, robust computational methods are necessary to facilitate statistical analysis. The processing of three‐dimensional data from eye tissue includes tasks such as data management, multichannel fluorescence image registration and fusion, and visualization and interactive analysis of the three‐dimensional image data. Several software tools contribute significantly to the comprehensive analysis of three‐dimensional images of eye tissue (Table 3).

**TABLE 3:** Statistical analysis software tool applied in eye optical clearing.

Software	Functionality	Application in eye tissue analysis
Vascular analysis software	• Quantifies vascular networks	Quantifies vessel density, length, and branching in retinal vasculature.
	• Calculates morphological parameters	

Comprehensive image processing software	• Channel manipulation	Analyzes spatial relationships between fluorescent labels and blood vessels.
	• Image quantification	

Advanced 3D imaging software	• 3D rendering and segmentation	Reconstructs ocular vasculature and neural networks in 3D; quantifies pathological changes.
	• Quantitative volume/density analysis	

Image analysis software development	• Automated segmentation	Enhances throughput and accuracy in large datasets (e.g., glaucoma or angiogenesis studies).
	• Dynamic parameter optimization	

#### 5.4.1. Vascular Analysis Software

Vascular analysis software facilitates fast, noninvasive, and reproducible quantification of vascular networks within images [69]. The software computes various morphological and spatial parameters, enabling swift analysis and statistical measurements of the retinal vascular network structure. Parameters such as the area covered by the vascular network, the number of vessels, vessel length, and vessel density can be quantified.

#### 5.4.2. Comprehensive Image Processing Software

Comprehensive image processing software can provide a range of common image processing functionalities, including format conversion, splitting and merging of imaging channels, image parameter adjustments, and image quantification [70]. When analyzing eye tissue, the comprehensive image processing software can be used for quantitative assessment of the vascular network. They can provide measurements such as vessel length, number of branching points, and outward growth of vessel‐like pseudopodia. Additionally, spatial relationships between fluorescent proteins and blood vessels can be analyzed using specific tools [71].

#### 5.4.3. Advanced 3D Imaging Software

This comprehensive software package is designed for the visualization and analysis of three‐dimensional imaging data. It encompasses modules for 3D rendering, video creation, image segmentation, and more. A specific functionality within the software allows for rendering the vascular system within eye tissue in three‐dimensional morphology, providing a realistic representation of the internal eye structure. Quantitative analysis can be conducted, including measurements of quantity, volume, density, and other aspects. These software tools enable analysis of discontinuous fluorescent protein signals with labels, as well as quantification of the branching hierarchy and angular characteristics of various vessel branches in the retinal vasculature. These specialized software tools for vascular and 3D imaging analysis are critical for extracting the rich information contained within 3D images of ocular tissues. They prove essential for unraveling intricate structural details, especially in the context of clinical, physiological, and pathological conditions [35].

## 6. Limitation and Future Perspective

Conventional histopathological analyses rely predominantly on two‐dimensional tissue sections, which are inherently prone to structural artifacts from sample preparation and may obscure critical three‐dimensional biological information. In contrast, whole‐tissue 3D histopathology offers superior spatial resolution and more comprehensive insights into anatomical and pathological relationships. Although tissue clearing techniques have revolutionized ocular research, several technical constraints remain to be addressed.

### 6.1. Current Main Challenges on Clearing Methods

Endogenous fluorescent proteins, such as GFP, are widely used in developmental and transgenic ocular studies [72, 73]. Preserving their signals is a key challenge in tissue clearing. Choosing the right clearing method is very critical. Organic solvent‐based methods, such as BABB, 3DISCO, and uDISCO, provide excellent transparency. However, they often cause fluorescence quenching. For example, ethanol dehydration in BABB reduces GFP signals. 3DISCO uses THF and DBE to better protect fluorescence, but DBE can degrade into peroxides and aldehydes, which harm fluorescent proteins. To solve this problem, PEGASOS and TESOS are alternatives that preserve endogenous fluorescence by creating a highly polar and peroxide‐free environment [18, 41]. In uDISCO, adding vitamin E prevents oxidation in the RI‐matching solution and extends signal lifetime [74, 75]. fDISCO uses a high‐pH environment to further stabilize GFP [76]. sDISCO added antioxidants to DBE, which improves fluorescence preservation [77].

A second challenge is autofluorescence and background signals. RPE cells contain lipofuscin, especially A2E (N‐retinyl idene‐N‐retinyl ethanolamine), which emits strong visible light fluorescence. This interferes with Enhanced Green Fluorescent Protein (eGFP), Enhanced Yellow Fluorescent Protein (eYFP), and mCherry signals. Fluorescent proteins are usually less bright than synthetic dyes, and clearing can further weaken their signals. Moreover, the bleaching process can also easily lead to the generation of autofluorescence (e.g., from heme or pigments). CuSO_4_ treatment and photobleaching with wide‐band visible light can also reduce autofluorescence in thick samples [78]. PEGASOS reduces A2E aggregation and promotes nonradiative decay, which lowers visible light autofluorescence in the RPE [18].

In general, tissue transformation‐based and high‐refractive index aqueous matching methods preserve endogenous fluorescence better than organic solvent‐based approaches. Aqueous matching methods are relatively gentle but often require long processing times and provide limited lipid removal, which may lead to tissue fragility. Tissue transformation‐based methods stabilize proteins and nucleic acids through crosslinking, thereby minimizing structural damage and fluorescence loss. CLARITY‐derived methods effectively preserve fluorescent signals but may compromise ultrastructure, particularly in lipid‐sparse ocular tissues [18, 45]. SHIELD (Stabilization under Harsh conditions via Intramolecular Epoxide Linkages to prevent Degradation) further enhances fluorescence and structural preservation in harsh chemical and thermal conditions [79]. PACT and psPACT are suitable for thicker samples, including large ocular tissues [80]. Emerging approaches such as VIVIT (vitreous ionic‐liquid‐solvent‐based volumetric inspection of trans‐scale biostructure) use glassy ionic liquid solvents. Because of their compatibility with delicate, water‐rich tissues, VIVIT‐based strategies may be promising for ocular clearing, although direct validation in eye tissues is still lacking [81].

A third challenge is tissue deformation [82]. Solvent‐based methods often cause tissue shrinkage due to strong dehydration. High‐refractive index aqueous matching methods may cause swelling. Tissue transformation‐based methods stabilize proteins and cells in a crosslinked network, which minimizes deformation. This is particularly important for studies of retinal vasculature or vitreoretinal interaction.

Finally, reagent toxicity is an important concern [78]. Solvent‐based methods use volatile organic chemicals such as THF and DBE, which are toxic and flammable [31]. High‐refractive index aqueous matching method reagents, including urea, sugars, and mild detergents, are much safer [43, 45]. Tissue transformation‐based methods use toxic monomers such as acrylamide during preparation, but the polymerized gel is inert and safe [49, 53]. Proper laboratory safety measures, such as working in a fume hood and wearing gloves and eye protection, are essential.

### 6.2. Clinical Translation Challenges in In Vivo Imaging

The eye’s unique optical properties permit noninvasive visualization of deep structures using modalities such as optical coherence tomography (OCT) and OCTA. However, conventional imaging systems face fundamental limitations in penetration depth and field of view, often generating artifacts in deep tissue imaging [83]. While modern tissue clearing techniques coupled with advanced imaging platforms now enable exquisite visualization of intraocular structures, most current protocols induce irreversible tissue alterations, severely restricting their clinical applicability.

Recently, tissue optical clearing techniques have garnered significant attention for their ability to render the skin and skull of living mice and rats transparent [84–88]. When combined with optical imaging systems, this approach enables high‐resolution visualization of cutaneous and deeper subdermal structures in vivo. The pioneering work by Kong et al. demonstrated the potential of reversible clearing using glycerol, achieving transient transparency of sclera, iris, and ciliary body in ex vivo rabbit eyes, with complete tissue reversion within 60 s postsaline rinse. Subsequently, OCT was used to observe the eye tissue, resulting in the acquisition of deeper tissue information [88]. The findings of this experiment offer valuable prospects for the application of eye tissue clearing techniques in live clinical diagnostics. However, it is crucial to acknowledge that the majority of currently available tissue clearing methods employ reagents that are harmful to the human body. Therefore, there is a need for the development of reversible, rapid, and safe eye tissue clearing techniques in the future, aiming to overcome the challenge of inadequate imaging capacity caused by light scattering within eye tissue. Such advancements would address the limitations faced by noninvasive detection devices. In a recent breakthrough, Ou and colleagues successfully implemented reversible optical clearing in live animals through the application of tartrazine, a strong absorptive molecule [87]. This research demonstrates significant potential for applications including corneal/lens transparency restoration, noninvasive imaging of fundus blood vessels, and observation of glaucoma drainage channels.

### 6.3. Overcoming Permeability Barriers in Ocular Tissue Clearing

State‐of‐the‐art tissue clearing methodologies, while capable of rendering various ocular components transparent, confront two major permeability‐related challenges. First, conventional pigment removal protocols employing hydrogen peroxide cause oxidative damage to cellular ultrastructure through oxygen radical generation during melanin bleaching. This underscores the urgent need for novel pigment‐clearing reagents that preserve molecular and structural integrity. More critically, the eye’s natural barriers—particularly the cornea and lens—severely restrict macromolecule penetration. Current workarounds involving corneal excision or intraocular injection inevitably induce structural perturbations while complicating experimental workflows. These limitations reveal a fundamental gap in clearing technology: the absence of effective strategies for enhancing reagent permeability across intact ocular barriers. Future innovations must focus on developing noninvasive permeability enhancers that can facilitate reagent diffusion without compromising tissue architecture, thereby enabling whole‐eye analysis with preserved native morphology.

### 6.4. Challenges in Preserving the Vitreous Body During Tissue Clearing

The vitreous humor and associated hyaloid and retinal vascular structures are central to many ocular developmental and pathological studies [89, 90]. However, preserving the vitreous during tissue clearing remains challenging. The vitreous has an extremely high water content and a unique extracellular matrix dominated by hyaluronan and sparse collagen fibers. These properties make it particularly vulnerable to standard clearing procedures. Most existing tissue clearing methods were not designed to preserve the vitreous body. Organic solvent‐based methods (such as DISCO‐based protocols) inevitably collapse the vitreous because of aggressive dehydration. In contrast, high‐refractive index aqueous matching methods often induce tissue swelling and destroy the collagen network. Tissue transformation‐based methods can partially stabilize ocular tissues, but they fail to adequately fix the vitreous due to its very low protein content. Several studies have attempted to address these limitations through protocol modifications. Nazari et al. adapted a 2,2′‐thiodiethanol (TDE) refractive index matching strategy to enable imaging of intact retina‐vitreous preparations [21]. Darche and colleagues applied iDISCO+ to albino mouse eyes to visualize regression of the vitreous vascular system [57]. Gurdita et al. further noted that dehydration‐based clearing strategies can cause substantial distortion of vitreous body. By contrast, hyperhydration‐based clearing methods such as CUBIC induces uniform tissue expansion, which can better approximate native ocular geometry. Building on this concept, Krimpenfort et al. modified depigmentation steps in CUBIC, ECi, and iDISCO+ protocols to preserve lens shape and thereby maintain vitreous vascular structures [13]. In addition, Yang et al. demonstrated that CLARITY enables comprehensive visualization of ocular components from the anterior to posterior segments, allowing discrimination of closely connected structures and assessment of pathological changes [13, 53].

### 6.5. Machine Learning–Enhanced Imaging and Analysis of Cleared Ocular Tissues

For imaging, since the eyeball comprises multiple tissue layers with varying optical properties and information content across each structure, optimal imaging requires adaptive scanning parameter adjustments during automated image acquisition. Such adaptive imaging can optimize signal‐to‐noise ratios and maximize image quality while minimizing scan time for each ocular structure. Looking ahead, integrating AI with imaging systems could enable intelligent skipping of noninformative image planes (e.g., blank areas) and dynamically optimize signal‐to‐noise ratios by providing structure‐specific imaging conditions throughout the entire process [91–94]. Moreover, current imaging systems face limitations due to restricted spectral channels, whereas ocular tissues comprise diverse cell types with complex molecular signatures. Expanding the spectral range of imaging devices could increase the number of detectable targets per scan by enabling multiplexed labeling [95]. For example, microscopy could include near‐infrared (800–1100 nm) or long‐wave infrared (1200–1600 nm) wavelengths. This advancement would significantly improve imaging throughput and diagnostic precision in a single acquisition.

For computational analysis, the eye’s complex vascular network and massive tissue‐clearing datasets make manual analysis extremely time‐consuming. Machine learning approaches can automate tedious steps, enhance image quality, and enable high‐fidelity 3D reconstruction of volumetric ocular images. Advanced AI‐based analysis can use convolutional neural networks to rapidly process 3D image signals and facilitate accurate segmentation and classification of cellular and subcellular structures [96, 97]. Together, these computational strategies can substantially reduce researcher workload and improve the efficiency and precision of ocular imaging studies.

## 7. Conclusion

Optical tissue clearing technology has revolutionized ocular research by enabling unprecedented 3D and even 4D visualization of previously inaccessible structures, such as the posterior eye’s lymphatic system. This breakthrough facilitates comprehensive analysis of vascular networks, neural circuits, and cellular components, while integrated AI enhances quantification accuracy, allowing for deeper investigation into disease mechanisms such as glaucoma and retinopathy. Current challenges include developing safer clinical protocols and improving antibody penetration. Future advancements promise to bridge the gap between research and clinical diagnostics, advancing both our understanding and treatment of ocular diseases. This transformative technology represents a paradigm shift in exploring ocular complexity, with profound implications for both scientific discovery and clinical applications.

## Author Contributions

Shuhuai Meng: writing–review and editing, writing–original draft, and conceptualization. Haiyang Sun: writing–review and editing, writing–original draft, and conceptualization. Zhengyi Xu: writing–original draft. Yixin Shi: writing–review and editing. Mingyang Chen: writing–review and editing. He Cai: writing–review and editing. Junyu Chen: writing–review and editing, supervision, funding acquisition, and conceptualization.

## Funding

This study was supported by the National Nature Science Foundation of China (Grant Nos. 82270961 and 82422021), Young Elite Scientist Sponsorship Program by CAST (Grant No. 2023QNRC001), and Sichuan Science and Technology Program (Grant No. 2023JDRC0018).

## Conflicts of Interest

The authors declare no conflicts of interest.

## References

[1] Zhang J. , Shi L. , and Shen Y. , The Retina: A Window in Which to View the Pathogenesis of Alzheimer’s Disease, Ageing Research Reviews. (2022) 77, 10.1016/j.arr.2022.101590. 35192959

[2] Gaire B. P. , Koronyo Y. , Fuchs D. T. et al., Alzheimer’s Disease Pathophysiology in the Retina, Progress in Retinal and Eye Research. (2024) 101, 10.1016/j.preteyeres.2024.101273. PMC11285518 38759947

[3] Boudriot E. , Stephan M. , Rabe F. et al., Genetic Analysis of Retinal Cell Types in Neuropsychiatric Disorders, JAMA Psychiatry. (2025) 82, no. 3, 285–295, 10.1001/jamapsychiatry.2024.4230. 39775833 PMC11883512

[4] Amini E. , Moghaddasi M. , Habibi S. A. H. et al., Huntington’s Disease and Neurovascular Structure of Retina, Neurological Sciences. (2022) 43, no. 10, 5933–5941, 10.1007/s10072-022-06232-3. 35771295

[5] Gouravani M. , Fekrazad S. , Mafhoumi A. , Ashouri M. , and DeBuc D. C. , Optical Coherence Tomography Measurements in Huntington’s Disease: A Systematic Review and Meta-Analysis, Journal of Neurology. (2024) 271, no. 10, 6471–6484, 10.1007/s00415-024-12634-4. 39187741 PMC11447008

[6] Lin J. B. and Apte R. S. , Seeing Parkinson Disease in the Retina, JAMA Ophthalmology. (2021) 139, no. 2, 189–190, 10.1001/jamaophthalmol.2020.5719. 33355611

[7] Fu C. , Yang N. , Chuang J. Z. et al., Mutant Mice With Rod-Specific VPS35 Deletion Exhibit Retinal α-Synuclein Pathology-Associated Degeneration, Nature Communications. (2024) 15, no. 1, 10.1038/s41467-024-50189-0. PMC11266608 39043666

[8] Fan J. , Rajapakse D. , Peterson K. et al., Retbindin Mediates Light-Damage in Mouse Retina While Its Absence Leads to Premature Retinal Aging, Experimental Eye Research. (2021) 209, 10.1016/j.exer.2021.108698. PMC8595511 34228964

[9] Asanad S. , Fantini M. , Sultan W. et al., Retinal Nerve Fiber Layer Thickness Predicts CSF Amyloid/Tau Before Cognitive Decline, PLoS One. (2020) 15, no. 5, 10.1371/journal.pone.0232785. PMC7259639 32469871

[10] Sparrow J. R. , Duncker T. , Schuerch K. , Paavo M. , and de Carvalho J. R. L.Jr., Lessons Learned From Quantitative Fundus Autofluorescence, Progress in Retinal and Eye Research. (2020) 74, 10.1016/j.preteyeres.2019.100774, 2-s2.0-85071421976. PMC7561015 31472235

[11] Gan Y. , Zhang X. , Su Y. , Shen M. , Peng Y. , and Wen F. , OCTA Versus Dye Angiography for the Diagnosis and Evaluation of Neovascularisation in Punctate Inner Choroidopathy, British Journal of Ophthalmology. (2022) 106, no. 4, 547–552, 10.1136/bjophthalmol-2020-318191. 33361443

[12] Lu W. , Wang Y. , Hu W. et al., A Novel Three-Dimensional Method for Detailed Analysis of RGC Central Projections Under Acute Ocular Hypertension, Experimental Eye Research. (2025) 250, 10.1016/j.exer.2024.110157. 39571780

[13] Krimpenfort L. T. , Garcia-Collado M. , van Leeuwen T. et al., Anatomy of the Complete Mouse Eye Vasculature Explored by Light-Sheet Fluorescence Microscopy Exposes Subvascular-Specific Remodeling in Development and Pathology, Experimental Eye Research. (2023) 237, 10.1016/j.exer.2023.109674. 37838300

[14] Darche M. , Borella Y. , Verschueren A. et al., Light Sheet Fluorescence Microscopy of Cleared Human Eyes, Communications Biology. (2023) 6, no. 1, 10.1038/s42003-023-05401-0. PMC10564773 37816868

[15] Tanaka Y. , Kubota A. , Yamato M. , Okano T. , and Nishida K. , Irreversible Optical Clearing of Sclera by Dehydration and Cross-Linking, Biomaterials. (2011) 32, no. 4, 1080–1090, 10.1016/j.biomaterials.2010.10.002, 2-s2.0-78649443361. 21055804

[16] Zaman R. T. , Rajaram N. , Nichols B. S. et al., Changes in Morphology and Optical Properties of Sclera and Choroidal Layers due to Hyperosmotic Agent, Journal of Biomedical Optics. (2011) 16, no. 7, 10.1117/1.3599985, 2-s2.0-80455141520. 21806288

[17] Tainaka K. , Murakami T. C. , Susaki E. A. et al., Chemical Landscape for Tissue Clearing Based on Hydrophilic Reagents, Cell Reports. (2018) 24, no. 8, 2196–2210.e9, 10.1016/j.celrep.2018.07.056, 2-s2.0-85051499044. 30134179

[18] Jing D. , Zhang S. , Luo W. et al., Tissue Clearing of Both Hard and Soft Tissue Organs With the PEGASOS Method, Cell Research. (2018) 28, no. 8, 803–818, 10.1038/s41422-018-0049-z, 2-s2.0-85047665337. 29844583 PMC6082844

[19] Manicam C. , Pitz S. , Brochhausen C. , Grus F. H. , Pfeiffer N. , and Gericke A. , Effective Melanin Depigmentation of Human and Murine Ocular Tissues: An Improved Method for Paraffin and Frozen Sections, PLoS One. (2014) 9, no. 7, 10.1371/journal.pone.0102512, 2-s2.0-84904269938. PMC4099143 25025426

[20] Kim S. Y. and Assawachananont J. , A New Method to Visualize the Intact Subretina From Retinal Pigment Epithelium to Retinal Tissue in Whole Mount of Pigmented Mouse Eyes, Translational Vision Science & Technology. (2016) 5, no. 1, 10.1167/tvst.5.1.6, 2-s2.0-85042811148. PMC4757471 26929886

[21] Nazari H. , Ivannikov M. , Ochoa L. , Vargas G. , and Motamedi M. , Microsurgical Dissection and Tissue Clearing for High Resolution Intact Whole Retina and Vitreous Imaging, Journal of Visualized Experiments. (2021) 169, 10.3791/61595. 33779596

[22] Esmer A. C. , Sivrikoz T. S. , Gulec E. Y. et al., Prenatal Diagnosis of Persistent Hyperplastic Primary Vitreous: Report of 2 Cases and Review of the Literature, Journal of Ultrasound in Medicine. (2016) 35, no. 10, 2285–2291, 10.7863/ultra.15.11040, 2-s2.0-84989328952. 27582535

[23] Richards A. J. , McNinch A. , Martin H. et al., Stickler Syndrome and the Vitreous Phenotype: Mutations in COL2A1 and COL11A1, Human Mutation. (2010) 31, no. 6, E1461–E1471, 10.1002/humu.21257, 2-s2.0-77952716339. 20513134

[24] Dodt H. U. , Leischner U. , Schierloh A. et al., Ultramicroscopy: Three-Dimensional Visualization of Neuronal Networks in the Whole Mouse Brain, Nature Methods. (2007) 4, no. 4, 331–336, 10.1038/nmeth1036, 2-s2.0-34247899223. 17384643

[25] Waxman S. , Loewen R. T. , Dang Y. , Watkins S. C. , Watson A. M. , and Loewen N. A. , High-Resolution, Three-Dimensional Reconstruction of the Outflow Tract Demonstrates Segmental Differences in Cleared Eyes, Investigative Ophthalmology & Visual Science. (2018) 59, no. 6, 2371–2380, 10.1167/iovs.17-23075, 2-s2.0-85046677212. 29847643 PMC5939687

[26] Stoecker A. , Pinkert-Leetsch D. , Koch T. et al., Collagen Crosslinking-Induced Corneal Morphological Changes: A Three-Dimensional Light Sheet Microscopy-Based Evaluation, Scientific Reports. (2024) 14, no. 1, 10.1038/s41598-024-78516-x. PMC11569206 39550403

[27] Kataoka H. , Ushiyama A. , Kawakami H. , Akimoto Y. , Matsubara S. , and Iijima T. , Fluorescent Imaging of Endothelial Glycocalyx Layer With Wheat Germ Agglutinin Using Intravital Microscopy, Microscopy Research and Technique. (2016) 79, no. 1, 31–37, 10.1002/jemt.22602, 2-s2.0-84954286375. 26768789

[28] Niu C. , Chen Z. , Kim K. T. et al., Metformin Alleviates Hyperglycemia-Induced Endothelial Impairment by Downregulating Autophagy via the Hedgehog Pathway, Autophagy. (2019) 15, no. 5, 843–870, 10.1080/15548627.2019.1569913, 2-s2.0-85060791765. 30653446 PMC6526809

[29] Lutty G. A. , McLeod D. S. , Bhutto I. A. , Edwards M. M. , and Seddon J. M. , Choriocapillaris Dropout in Early Age-Related Macular Degeneration, Experimental Eye Research. (2020) 192, 10.1016/j.exer.2020.107939. PMC7216757 31987759

[30] Zhao S. , Todorov M. I. , Cai R. et al., Cellular and Molecular Probing of Intact Human Organs, Cell. (2020) 180, no. 4, 796–812.e19, 10.1016/j.cell.2020.01.030. 32059778 PMC7557154

[31] Ertürk A. , Becker K. , Jährling N. et al., Three-Dimensional Imaging of Solvent-Cleared Organs Using 3DISCO, Nature Protocols. (2012) 7, no. 11, 1983–1995, 10.1038/nprot.2012.119, 2-s2.0-84868135888. 23060243

[32] Drake S. S. , Charabati M. , Simas T. et al., 3-Dimensional Immunostaining and Automated Deep-Learning Based Analysis of Nerve Degeneration, International Journal of Molecular Sciences. (2022) 23, no. 23, 10.3390/ijms232314811. PMC9739543 36499143

[33] Bray E. R. , Noga M. , Thakor K. et al., 3D Visualization of Individual Regenerating Retinal Ganglion Cell Axons Reveals Surprisingly Complex Growth Paths, eNeuro. (2017) 4, no. 4, 10.1523/eneuro.0093-17.2017, 2-s2.0-85032169480. PMC5575138 28856242

[34] Renier N. , Adams E. L. , Kirst C. et al., Mapping of Brain Activity by Automated Volume Analysis of Immediate Early Genes, Cell. (2016) 165, no. 7, 1789–1802, 10.1016/j.cell.2016.05.007, 2-s2.0-84973441068. 27238021 PMC4912438

[35] Henning Y. , Osadnik C. , and Malkemper E. P. , EyeCi: Optical Clearing and Imaging of Immunolabeled Mouse Eyes Using Light-Sheet Fluorescence Microscopy, Experimental Eye Research. (2019) 180, 137–145, 10.1016/j.exer.2018.12.001, 2-s2.0-85059129517. 30578790

[36] Vigouroux R. J. , Cesar Q. , Chédotal A. , and Nguyen-Ba-Charvet K. T. , Revisiting the Role of Dcc in Visual System Development With a Novel Eye Clearing Method, eLife. (2020) 9, 10.7554/elife.51275. PMC7062470 32096760

[37] Mai H. , Luo J. , Hoeher L. et al., Whole-Body Cellular Mapping in Mouse Using Standard IgG Antibodies, Nature Biotechnology. (2024) 42, no. 4, 617–627, 10.1038/s41587-023-01846-0. PMC11021200 37430076

[38] Luo J. , Molbay M. , Chen Y. et al., Nanocarrier Imaging at Single-Cell Resolution Across Entire Mouse Bodies With Deep Learning, Nature Biotechnology. (2025) 43, no. 12, 2009–2022, 10.1038/s41587-024-02528-1. PMC12700832 39809933

[39] Nudell V. , Wang Y. , Pang Z. et al., HYBRiD: Hydrogel-Reinforced DISCO for Clearing Mammalian Bodies, Nature Methods. (2022) 19, no. 4, 479–485, 10.1038/s41592-022-01427-0. 35347322 PMC9337799

[40] Renier N. , Wu Z. , Simon D. , Yang J. , Ariel P. , and Tessier-Lavigne M. , iDISCO: A Simple, Rapid Method to Immunolabel Large Tissue Samples for Volume Imaging, Cell. (2014) 159, no. 4, 896–910, 10.1016/j.cell.2014.10.010, 2-s2.0-84910092406. 25417164

[41] Yi Y. , Li Y. , Zhang S. et al., Mapping of Individual Sensory Nerve Axons From Digits to Spinal Cord With the Transparent Embedding Solvent System, Cell Research. (2024) 34, no. 2, 124–139, 10.1038/s41422-023-00867-3. 38168640 PMC10837210

[42] Denk W. , Strickler J. H. , and Webb W. W. , Two-Photon Laser Scanning Fluorescence Microscopy, Science. (1990) 248, no. 4951, 73–76, 10.1126/science.2321027, 2-s2.0-0025342635. 2321027

[43] Ke M. T. , Fujimoto S. , and Imai T. , SeeDB: A Simple and Morphology-Preserving Optical Clearing Agent for Neuronal Circuit Reconstruction, Nature Neuroscience. (2013) 16, no. 8, 1154–1161, 10.1038/nn.3447, 2-s2.0-84880920117. 23792946

[44] Hohberger B. , Baumgart C. , and Bergua A. , Optical Clearing of the Eye Using the See Deep Brain Technique, Eye. (2017) 31, no. 10, 1496–1502, 10.1038/eye.2017.83, 2-s2.0-85031321262. 28574496 PMC5639196

[45] Susaki E. A. , Tainaka K. , Perrin D. et al., Whole-Brain Imaging With Single-Cell Resolution Using Chemical Cocktails and Computational Analysis, Cell. (2014) 157, no. 3, 726–739, 10.1016/j.cell.2014.03.042, 2-s2.0-84899620303. 24746791

[46] Matsumoto K. , Mitani T. T. , Horiguchi S. A. et al., Advanced CUBIC Tissue Clearing for Whole-Organ Cell Profiling, Nature Protocols. (2019) 14, no. 12, 3506–3537, 10.1038/s41596-019-0240-9. 31748753

[47] Ye Y. , Dinh Duong T. A. , Saito K. et al., Visualization of the Retina in Intact Eyes of Mice and Ferrets Using a Tissue Clearing Method, Translational Vision Science & Technology. (2020) 9, no. 3, 10.1167/tvst.9.3.1. PMC7347279 32704421

[48] Jing D. , Yi Y. , Luo W. et al., Tissue Clearing and Its Application to Bone and Dental Tissues, Journal of Dental Research. (2019) 98, no. 6, 621–631, 10.1177/0022034519844510, 2-s2.0-85064837063. 31009584 PMC6535919

[49] Chung K. , Wallace J. , Kim S. Y. et al., Structural and Molecular Interrogation of Intact Biological Systems, Nature. (2013) 497, no. 7449, 332–337, 10.1038/nature12107, 2-s2.0-84878115127. 23575631 PMC4092167

[50] Alessio E. J. and Zhang D. Q. , Immunostaining of Whole-Mount Retinas With the CLARITY Tissue Clearing Method, Journal of Visualized Experiments. (2021) 10.3791/62178. 33749674

[51] Chang C. C. , Chu A. , Meyer S. et al., Three-Dimensional Imaging Coupled With Topological Quantification Uncovers Retinal Vascular Plexuses Undergoing Obliteration, Theranostics. (2021) 11, no. 3, 1162–1175, 10.7150/thno.53073. 33391527 PMC7738897

[52] Singh J. N. , Nowlin T. M. , Seedorf G. J. , Abman S. H. , and Shepherd D. P. , Quantifying Three-Dimensional Rodent Retina Vascular Development Using Optical Tissue Clearing and Light-Sheet Microscopy, Journal of Biomedical Optics. (2017) 22, no. 7, 10.1117/1.jbo.22.7.076011, 2-s2.0-85025806390. PMC5514054 28717817

[53] Yang Y. , Li G. , and Chen L. , High Resolution Three-Dimensional Imaging of the Ocular Surface and Intact Eyeball Using Tissue Clearing and Light Sheet Microscopy, Ocular Surface. (2020) 18, no. 3, 526–532, 10.1016/j.jtos.2020.04.009. 32417103 PMC8099854

[54] Yang B. , Treweek J. , Kulkarni R. et al., Single-Cell Phenotyping Within Transparent Intact Tissue Through Whole-Body Clearing, Cell. (2014) 158, no. 4, 945–958, 10.1016/j.cell.2014.07.017, 2-s2.0-84908404143. 25088144 PMC4153367

[55] Prahst C. , Ashrafzadeh P. , Mead T. et al., Mouse Retinal Cell Behaviour in Space and Time Using Light Sheet Fluorescence Microscopy, eLife. (2020) 9, 10.7554/elife.49779. PMC7162655 32073398

[56] Cora V. , Haderspeck J. , Antkowiak L. et al., A Cleared View on Retinal Organoids, Cells. (2019) 8, no. 5, 10.3390/cells8050391. PMC6562974 31035373

[57] Darche M. , Verschueren A. , Belle M. et al., Three-Dimensional Characterization of Developing and Adult Ocular Vasculature in Mice Using in Toto Clearing, Communications Biology. (2022) 5, no. 1, 10.1038/s42003-022-04104-2. PMC9613908 36302949

[58] Yin X. , Zhang S. , Lee J. H. et al., Compartmentalized Ocular Lymphatic System Mediates Eye-Brain Immunity, Nature. (2024) 628, no. 8006, 204–211, 10.1038/s41586-024-07130-8. 38418880 PMC10990932

[59] Cooper M. L. , Gildea H. K. , Selles M. C. , Katafygiotou E. , Liddelow S. A. , and Chao M. V. , Astrocytes in the Mouse Brain Respond Bilaterally to Unilateral Retinal Neurodegeneration, Proceedings of the National Academy of Sciences of the United States of America. (2025) 122, no. 11, 10.1073/pnas.2418249122. PMC11929491 40063795

[60] Yu D. Y. , Cringle S. J. , Yu P. K. et al., Retinal Capillary Perfusion: Spatial and Temporal Heterogeneity, Progress in Retinal and Eye Research. (2019) 70, 23–54, 10.1016/j.preteyeres.2019.01.001, 2-s2.0-85061825450. 30769149

[61] Moos F. , Suppinger S. , de Medeiros G. et al., Open-Top Multisample Dual-View Light-Sheet Microscope for Live Imaging of Large Multicellular Systems, Nature Methods. (2024) 21, no. 5, 798–803, 10.1038/s41592-024-02213-w. 38509326 PMC11093739

[62] Otomo K. , Omura T. , Nozawa Y. et al., descSPIM: An Affordable and Easy-to-Build Light-Sheet Microscope Optimized for Tissue Clearing Techniques, Nature Communications. (2024) 15, no. 1, 10.1038/s41467-024-49131-1. PMC11169475 38866781

[63] Santos-Durán G. N. , Cooper R. L. , Jahanbakhsh E. , Timin G. , and Milinkovitch M. C. , Self-Organized Patterning of Crocodile Head Scales by Compressive Folding, Nature. (2025) 637, no. 8045, 375–383, 10.1038/s41586-024-08268-1. 39663449 PMC11711089

[64] Reynaud E. G. , Peychl J. , Huisken J. , and Tomancak P. , Guide to Light-Sheet Microscopy for Adventurous Biologists, Nature Methods. (2015) 12, no. 1, 30–34, 10.1038/nmeth.3222, 2-s2.0-84925033174. 25549268

[65] Stahl A. , Connor K. M. , Sapieha P. et al., The Mouse Retina as an Angiogenesis Model, Investigative Ophthalmology & Visual Science. (2010) 51, no. 6, 2813–2826, 10.1167/iovs.10-5176, 2-s2.0-77953244680. 20484600 PMC2891451

[66] Tseng S. J. , Lee Y. H. , Chen Z. H. , Lin H. H. , Lin C. Y. , and Tang S. C. , Integration of Optical Clearing and Optical Sectioning Microscopy for Three-Dimensional Imaging of Natural Biomaterial Scaffolds in Thin Sections, Journal of Biomedical Optics. (2009) 14, no. 4, 10.1117/1.3158998, 2-s2.0-73349118498. 19725716

[67] Sdobnov A. Y. , Darvin M. , Genina E. , Bashkatov A. , Lademann J. , and Tuchin V. , Recent Progress in Tissue Optical Clearing for Spectroscopic Application, Spectrochimica Acta Part A: Molecular and Biomolecular Spectroscopy. (2018) 197, 216–229, 10.1016/j.saa.2018.01.085, 2-s2.0-85041724196. 29433855

[68] Lee J. H. , Lee S. , Gho Y. S. et al., Comparison of Confocal Microscopy and Two-Photon Microscopy in Mouse Cornea In Vivo, Experimental Eye Research. (2015) 132, 101–108, 10.1016/j.exer.2015.01.013, 2-s2.0-84922683308. 25602499

[69] Zudaire E. , Gambardella L. , Kurcz C. , and Vermeren S. , A Computational Tool for Quantitative Analysis of Vascular Networks, PLoS One. (2011) 6, no. 11, 10.1371/journal.pone.0027385, 2-s2.0-81155134376. PMC3217985 22110636

[70] Schindelin J. , Arganda-Carreras I. , Frise E. et al., Fiji: An Open-Source Platform for Biological-Image Analysis, Nature Methods. (2012) 9, no. 7, 676–682, 10.1038/nmeth.2019, 2-s2.0-84862520770. 22743772 PMC3855844

[71] Segarra M. , Aburto M. R. , Cop F. et al., Endothelial Dab1 Signaling Orchestrates Neuro-Glia-Vessel Communication in the Central Nervous System, Science. (2018) 361, no. 6404, 10.1126/science.aao2861, 2-s2.0-85052119530. 30139844

[72] Shen J. , Lima e Silva R. , Zhang M. et al., Suprachoroidal Gene Transfer With Nonviral Nanoparticles in Large Animal Eyes, Science Advances. (2024) 10, no. 10, 10.1126/sciadv.adl3576. PMC10923522 38457512

[73] Scruggs B. A. , Berger A. , Knudsen T. et al., Retinal Gene Therapy Using Epiretinal AAV-Containing Fibrin Hydrogel Implants, Science Advances. (2025) 11, no. 36, 10.1126/sciadv.adv7922. PMC12412667 40911667

[74] Chen L. , Meng J. , Zhou Y. et al., Efficient 3D Imaging and Pathological Analysis of the Human Lymphoma Tumor Microenvironment Using Light-Sheet Immunofluorescence Microscopy, Theranostics. (2024) 14, no. 1, 406–419, 10.7150/thno.86221. 38164148 PMC10750216

[75] Pan C. , Cai R. , Quacquarelli F. P. et al., Shrinkage-Mediated Imaging of Entire Organs and Organisms Using uDISCO, Nature Methods. (2016) 13, no. 10, 859–867, 10.1038/nmeth.3964, 2-s2.0-84983491117. 27548807

[76] Qi Y. , Yu T. , Xu J. et al., FDISCO: Advanced Solvent-Based Clearing Method for Imaging Whole Organs, Science Advances. (2019) 5, no. 1, 10.1126/sciadv.aau8355, 2-s2.0-85060053692. PMC6357753 30746463

[77] Pichardo A. H. , Amadeo F. , Wilm B. et al., Optical Tissue Clearing to Study the Intra-Pulmonary Biodistribution of Intravenously Delivered Mesenchymal Stromal Cells and Their Interactions With Host Lung Cells, International Journal of Molecular Sciences. (2022) 23, no. 22, 10.3390/ijms232214171. PMC9699424 36430651

[78] Molbay M. , Kolabas Z. I. , Todorov M. I. , Ohn T. , and Ertürk A. , A Guidebook for DISCO Tissue Clearing, Molecular Systems Biology. (2021) 17, no. 3, 10.15252/msb.20209807. PMC7995442 33769689

[79] Park Y. G. , Sohn C. H. , Chen R. et al., Protection of Tissue Physicochemical Properties Using Polyfunctional Crosslinkers, Nature Biotechnology. (2019) 37, 73–83. 10.1038/nbt.4281 PMC6579717 30556815

[80] Treweek J. B. , Chan K. Y. , Flytzanis N. C. et al., Whole-Body Tissue Stabilization and Selective Extractions via Tissue-Hydrogel Hybrids for High-Resolution Intact Circuit Mapping and Phenotyping, Nature Protocols. (2015) 10, no. 11, 1860–1896, 10.1038/nprot.2015.122, 2-s2.0-84965191403. 26492141 PMC4917295

[81] Gao Y. , Xin F. , Wang T. et al., VIVIT: Resolving Trans-Scale Volumetric Biological Architectures via Ionic Glassy Tissue, Cell. (2025) 188, no. 21, 6079–6095.e20, 10.1016/j.cell.2025.07.023. 40795853

[82] Wan P. , Zhu J. , Xu J. , Li Y. , Yu T. , and Zhu D. , Evaluation of Seven Optical Clearing Methods in Mouse Brain, Neurophotonics. (2018) 5, no. 3, 10.1117/1.nph.5.3.035007, 2-s2.0-85052943948. PMC6109056 30155510

[83] Kashani A. H. , Asanad S. , Chan J. W. et al., Past, Present and Future Role of Retinal Imaging in Neurodegenerative Disease, Progress in Retinal and Eye Research. (2021) 83, 10.1016/j.preteyeres.2020.100938. PMC8280255 33460813

[84] Yu T. , Li D. , and Zhu D. , Tissue Optical Clearing for Biomedical Imaging: From In Vitro to In Vivo, Advances in Experimental Medicine and Biology. (2021) 3233, 217–255. 34053030 10.1007/978-981-15-7627-0_11

[85] Li D. Y. , Zheng Z. , Yu T. et al., Visible-Near Infrared-II Skull Optical Clearing Window for In Vivo Cortical Vasculature Imaging and Targeted Manipulation, Journal of Biophotonics. (2020) 13, no. 10, 10.1002/jbio.202000142. 32589789

[86] Bragina O. A. , Atochin D. A. , Trofimov A. O. , Nemoto E. , and Bragin D. E. , Cerebral Microcirculation and Oxygenation Modulation by Transcranial Alternating Current Stimulation in Awake and Anesthetized Mice, Advances in Experimental Medicine and Biology. (2023) 1438, 9–13. 37845432 10.1007/978-3-031-42003-0_2 PMC11354134

[87] Ou Z. , Duh Y. S. , Rommelfanger N. J. et al., Achieving Optical Transparency in Live Animals With Absorbing Molecules, Science. (2024) 385, no. 6713, 10.1126/science.adm6869. PMC11931656 39236186

[88] Kong R. , Wu W. , Qiu R. et al., Imaging Depth Extension of Optical Coherence Tomography in Rabbit Eyes Using Optical Clearing Agents, Experimental Biology and Medicine. (2020) 245, no. 18, 1629–1636, 10.1177/1535370220949834. 32791848 PMC7802378

[89] Lutty G. A. and McLeod D. S. , Development of the Hyaloid, Choroidal and Retinal Vasculatures in the Fetal Human Eye, Progress in Retinal and Eye Research. (2018) 62, 58–76, 10.1016/j.preteyeres.2017.10.001, 2-s2.0-85032947018. 29081352 PMC5776052

[90] Yoshikawa Y. , Yamada T. , Tai-Nagara I. et al., Developmental Regression of Hyaloid Vasculature is Triggered by Neurons, Journal of Experimental Medicine. (2016) 213, no. 7, 1175–1183, 10.1084/jem.20151966, 2-s2.0-84977632685. 27325890 PMC4925022

[91] Mahecic D. , Stepp W. L. , Zhang C. , Griffié J. , Weigert M. , and Manley S. , Event-Driven Acquisition for Content-Enriched Microscopy, Nature Methods. (2022) 19, no. 10, 1262–1267, 10.1038/s41592-022-01589-x. 36076039 PMC7613693

[92] Alvelid J. , Damenti M. , Sgattoni C. , and Testa I. , Event-Triggered STED Imaging, Nature Methods. (2022) 19, no. 10, 1268–1275, 10.1038/s41592-022-01588-y. 36076037 PMC9550628

[93] Shi Y. , Tabet J. S. , Milkie D. E. et al., Smart Lattice Light-Sheet Microscopy for Imaging Rare and Complex Cellular Events, Nature Methods. (2024) 21, no. 2, 301–310, 10.1038/s41592-023-02126-0. 38167656 PMC11216155

[94] Lin T. , Wang M. , Lin A. et al., Efficiency and Safety of Automated Label Cleaning on Multimodal Retinal Images, NPJ Digital Medicine. (2025) 8, no. 1, 10.1038/s41746-024-01424-x. PMC11701072 39757295

[95] Shi L. , Wei M. , Miao Y. et al., Highly-Multiplexed Volumetric Mapping With Raman Dye Imaging and Tissue Clearing, Nature Biotechnology. (2022) 40, no. 3, 364–373, 10.1038/s41587-021-01041-z. PMC8930416 34608326

[96] Li X. , Li Y. , Zhou Y. et al., Real-Time Denoising Enables High-Sensitivity Fluorescence Time-Lapse Imaging Beyond the Shot-Noise Limit, Nature Biotechnology. (2023) 41, no. 2, 282–292, 10.1038/s41587-022-01450-8. PMC9931589 36163547

[97] Bouchard C. , Bernatchez R. , and Lavoie-Cardinal F. , Addressing Annotation and Data Scarcity When Designing Machine Learning Strategies for Neurophotonics, Neurophotonics. (2023) 10, no. 4, 10.1117/1.nph.10.4.044405. PMC10447257 37636490

